# Ultra-Deep Pyrosequencing Detects Conserved Genomic Sites and Quantifies Linkage of Drug-Resistant Amino Acid Changes in the Hepatitis B Virus Genome

**DOI:** 10.1371/journal.pone.0037874

**Published:** 2012-05-30

**Authors:** Francisco Rodriguez-Frías, David Tabernero, Josep Quer, Juan I. Esteban, Israel Ortega, Esteban Domingo, Maria Cubero, Sílvia Camós, Carles Ferrer-Costa, Alex Sánchez, Rosendo Jardí, Melanie Schaper, Maria Homs, Damir Garcia-Cehic, Jaume Guardia, Rafael Esteban, Maria Buti

**Affiliations:** 1 Biochemistry Department, Hospital Universitari Vall d'Hebron (HUVH), Vall d'Hebron Institut de Recerca (VHIR), Universitat Autònoma de Barcelona (UAB), Barcelona, Spain; 2 Centro de Investigación Biomédica en Red de Enfermedades Hepáticas y Digestivas (CIBERehd), Instituto de Salud Carlos III, Madrid, Spain; 3 Liver Unit, Hospital Universitari Vall d'Hebron (HUVH),Vall d'Hebron Institut de Recerca (VHIR), Universitat Autònoma de Barcelona (UAB), Barcelona, Spain; 4 Unitat d'Estadística i Bioinformàtica (UEB), Hospital Universitari Vall d'Hebron (HUVH),Vall d'Hebron Institut de Recerca (VHIR), Universitat Autonoma de Barcelona (UAB), Barcelona, Spain; 5 Centro Biologia Molecular “Severo Ochoa” (CBMSO), CSIC-Universidad Autonoma de Madrid (UAM), Madrid, Spain; 6 Molecular Modelling and Bioinformatics Unit, Structural Biology Node, Institut de Recerca Biomèdica (IRB), Parc Científic de Barcelona, Barcelona, Spain; 7 Departament d'Estadística, Facultat Biologia, Universitat de Barcelona (UB), Barcelona, Spain; Saint Louis University, United States of America

## Abstract

**Background:**

Selection of amino acid substitutions associated with resistance to nucleos(t)ide-analog (NA) therapy in the hepatitis B virus (HBV) reverse transcriptase (RT) and their combination in a single viral genome complicates treatment of chronic HBV infection and may affect the overlapping surface coding region. In this study, the variability of an overlapping polymerase-surface region, critical for NA resistance, is investigated before treatment and under antiviral therapy, with assessment of NA-resistant amino acid changes simultaneously occurring in the same genome (linkage analysis) and their influence on the surface coding region.

**Methodology/Principal Findings:**

Serum samples obtained from chronic HBV-infected patients at pre-treatment and during sequential NA treatment with lamivudine, adefovir, and entecavir were analyzed by ultra-deep pyrosequencing (UDPS) using the GS-FLX platform (454 Life Sciences-Roche). The pre-treatment HBV quasispecies was not enriched with NA-resistant substitutions. The frequencies of this type of substitutions at pre-treatment did not predict the frequencies observed during lamivudine treatment. On linkage analysis of the RT region studied, NA-resistant HBV variants (except for rtA181T) were present in combinations of amino acid substitutions that increased in complexity after viral breakthrough to entecavir, at which time the combined variant rtL180M-S202G-M204V-V207I predominated. In the overlapping surface region, NA-resistant substitutions caused selection of stop codons in a significant percentage of sequences both at pre-treatment and during sequential treatment; the rtA181T substitution, related to sW172stop, predominated during treatment with lamivudine and adefovir. A highly conserved RT residue (rtL155), even more conserved than the essential residues in the RT catalytic motif YMDD, was identified in all samples.

**Conclusions:**

UDPS methodology enabled quantification of HBV quasispecies variants, even those harboring complex combinations of amino acid changes. The high percentage of potentially defective genomes, especially in the surface region, suggests effective trans-complementation of these variants.

## Introduction

The hepatitis B virus (HBV) is a DNA virus that replicates via reverse transcription of an RNA intermediate [1] with rapid viral turnover (>10^11^ virions per day) [2] and high mutation rates (>10^−5^ nucleotide substitutions/site/year) [3], despite the extremely high overlap of its coding regions [4]. Therefore, the virus circulates as a complex quasispecies, undergoing continuous changes due to competition and interactions between the genetic variants produced [5]. Chronic HBV infection (CHB) affects 350 million people worldwide and is a leading cause of liver cirrhosis and hepatocellular carcinoma [6]. Currently, five oral nucleos(t)ide analogues (NAs) are approved for CHB treatment in many parts of the world: lamivudine (LMV), adefovir-dipivoxil (ADV), entecavir (ETV), telbivudine (LdT) and tenofovir (TDF) [6], [7]. These drugs prevent viral replication by inhibiting the HBV reverse transcriptase (RT), but do not avoid formation of the covalently closed circular DNA (cccDNA), the main intracellular intermediate of HBV which is responsible for viral persistence, and they clear it at very slow rates [7]–[9]. Therefore long-term treatment is needed to maintain suppression of replication [8], [9]. These therapies may result in selection of antiviral-resistant HBV variants from the HBV quasispecies pool over time, leading to treatment failure and disease progression. In treatment-naïve patients, selection of NA-resistant variants is highly probable with LMV, intermediate with ADV and LdT, and minimal or even zero with ETV and TDF, which are currently recommended as first-choice therapies against CHB [6], [7].

Currently, identification of HBV genotypic NA resistance is mainly performed by PCR amplification and Sanger sequencing or the INNO-LiPA line probe reverse hybridization assay (LiPA), which detect NA resistance amino acid (aa) substitutions present in the HBV quasispecies in proportions of 20% and between 5%–10%, respectively [7]. With these methods the frequencies of each mutation cannot be measured, and it is impossible to determine whether several mutations are combined in the same sequence (mutation linkage). This is an important limitation because the addition of compensatory or secondary NA resistance aa substitutions to primary resistance substitutions in NA-resistant genomes (which usually reduce their replication efficiency in relation to wild-type variants), may enhance the replication of these genomes or even give rise to multidrug resistant (MDR) variants [10], [11]. As an alternative to LiPA and direct sequencing, molecular cloning enables analysis of single DNA molecules, thereby surpassing the detection limits of the other techniques. Nevertheless, this methodology is complex and time-consuming because analysis of at least 100 clones is required to detect variants present in 1% of the quasispecies. These limitations can now be overcome by ultra-deep pyrosequencing (UDPS) technology based on the GS-FLX platform (454 Life Sciences-Roche), which enables quantitative analysis of thousands of clonally amplified sequences [12] up to 400 nucleotides (nts) in length. With this technique, minor variants in the HBV quasispecies can be detected and quantified, including those with combinations of changes in the same sequence. In HIV infection, UDPS has been used to detect clinically relevant minority drug-resistance mutants with much higher sensitivity (<1%) than cloning [13], [14]. It has been suggested that quantification of minor drug-resistance mutants in treatment-experienced HIV-infected patients may be helpful when planning subsequent antiretroviral regimens [15]. A similar approach has been used to analyze HBV quasispecies in the RT and the overlapping hepatitis B surface antigen (HBsAg) coding regions in drug-naive (untreated) and drug-resistant patients [16], [17]. However, the simultaneous presence of various NA resistance-related aa substitutions in the same genome was not analyzed.

**Figure 1:**
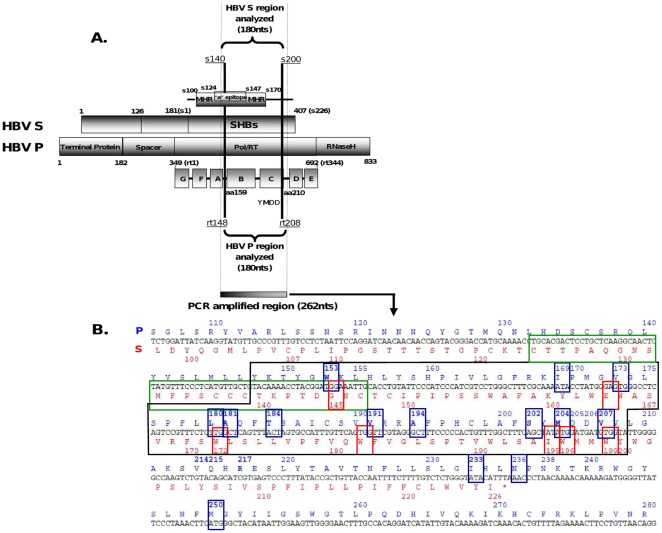
Fragment of HBV genome analyzed by UDPS. **A.** Initially a 224-bp fragment of the HBV genome was amplified with the fusion primers, obtaining a 262-bp amplicon. After trimming both primer ends, a 180-nucleotide fragment was analyzed. The fragment contains the B and C domains of the HBV RT in the polymerase ORF, spanning rtY148 to rtV208, and a region of the overlapping surface ORF, from sT140 to sY200, including a part of the main hydrophilic region, which contains a small portion of the C-terminus of the immunodominant epitope “a determinant”, framed in green in the sequence below. **B.** The sequence framed in black corresponds to that of the 180-nucleotide fragment analyzed and shows its translation into aa in the P (above, depicted in blue) and S (below, depicted in red) ORFs. Most of the main codons related to nucleos(t)ide resistance (framed in blue), and the overlapping codons in the S ORF that may give rise to immune escape or stop codons (framed in red) are located within the analyzed fragment. Codons rt233, rt236, and rt250 were not considered relevant for the aims of this study and were not analyzed.

In the present study, a coding region where HBV polymerase (P) and surface (S) open reading frames (ORFs) overlap was clonally analyzed by UDPS with standard GS-FLX technology (the only method available when the study was designed), which allows sequencing of DNA fragments up to 250 bp in length, including the fusion primers (see Patients and Methods). The region analyzed extends from codon rt148 to rt208 in the P ORF, which includes most RT codons corresponding to reported relevant NA-resistant aa substitutions (rtI169T, rtV173L, rtL180M, rtA181T/V, rtT184S/A/I/L/G/C/M, rtA194T, rtA202C/G/I, rtM204V/I [7], rtW153Q [18], rtV191I [19] and rtV207I [20]), and spans a part of the S ORF, from codon s140 to s200, containing the C-terminal region of the immunodominant epitope “a determinant” (codon s124 to s147) (Figure 1). This was done to exploit the capability of UDPS technology to quantify variants in extremely low percentages in the viral quasispecies in order to study the variability of the overlapping coding region before NA treatment and during sequential treatment. Our rationale for performing this research was that in the absence of antiviral treatment, ultra-deep analysis of the variability in this region might identify conserved positions in the RT region with a potentially essential functional role in the HBV polymerase and would be helpful to establish whether the percentage of HBV NA-resistant variants in the baseline quasispecies could predict their subsequent selection by a particular NA treatment. Furthermore, during sequential NA treatment, the ability of UDPS to analyze clonally amplified sequences would enable study of the simultaneous presence of various aa substitutions (linkage analysis) related to treatment failure in the same genome and detection of enrichment in MDR variants. This is of particular relevance in LMV resistance, which increases the probability of developing cross-resistance to subsequent treatments with ETV or ADV [21]–[24]. In addition, enrichment of the HBV quasispecies in MDR variants could result in changes in epitopic regions of HBsAg that could potentially affect virion assembly, stability or infectivity, owing to the complete overlap between the P and S ORFs [4], [25]. Thus, aa substitutions present in the S ORF were also studied to determine the influence of MDR variant enrichment.

## Results

A total of 350 744 reads from 8 serum samples were obtained after filtering the UDPS results with the GS-Amplicon-Variant-Analysis software included in GS-FLX platform. To increase the sensitivity and accuracy of UDPS, these reads were additionally filtered by an automated computational algorithm that excluded those likely to contain artifactual single-base changes based on Poisson distribution [13]. By this approach, 245 565 reads (13 670 to 62 450 per sample) were validated. In each sample, the most highly represented nucleotide sequence was designated “master”. Alignment between these master sequences is shown in Dataset S1, and translations into amino acids in the P and S ORFs are shown in Dataset S2 and Dataset S3, respectively.

### UDPS analysis of pre-treatment patient samples

**Figure 2:**
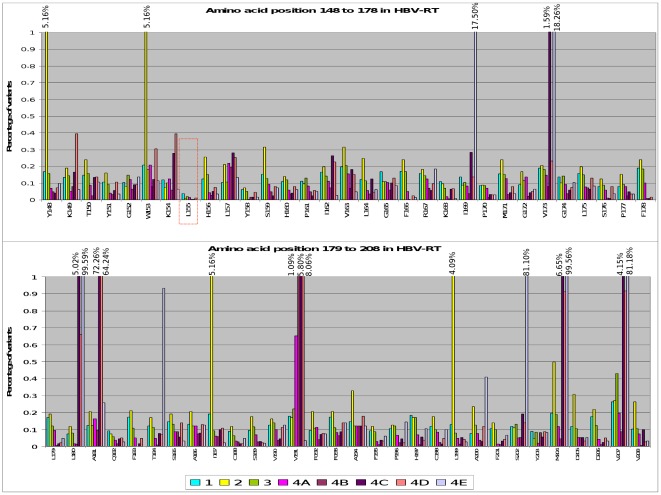
Overall frequency of amino acid substitutions at each reverse transcriptase position. Each bar represents the sum of frequencies of all amino acid changes occurring in each reverse transcriptase position. Each bar with a different color in the same position corresponds to one of the 8 samples studied in the 4 patients. Among these positions rtL155 (framed in red) was the least variable. Samples 1, 2, 3, 4A and 4B were obtained prior to antiviral treatment, whereas samples 4C, 4D and 4E correspond to completion of lamivudine, adefovir, and entecavir treatments, respectively.

A total of 141 581 validated reads were obtained from the 4 pre-treatment patient samples (1, 2, 3 and 4A). In each sample, the master sequence matched the consensus sequence obtained by Sanger method. Each validated read was aligned with the master sequence from the corresponding sample to calculate the frequency of each aa change relative to the master. Using this method, we found that the frequency of aa substitutions associated with NA treatment resistance was not significantly different from that of the remaining aa changes; the differences approached significance only in patient 4 (*P* = 0.09) (Table 1). On calculation of the average percentage of each aa change in the 4 baseline populations, 13 changes were found at average frequencies of ≥0.1% (Table S1), and 4 of them are known to confer NA resistance: rtA181T (0.10%), rtV191I (0.23%), rtA194T (0.11%), and rtM204I (0.15%). The dominant sequence in patient 2 was accompanied by around 5% of sequences carrying substitutions rtH148Y, rtR153W, rtI187L and rtL199V (Figure 2). In addition, substitutions rtD205N and rtD206N, both of which affect essential residues from the YMDD catalytic motif, showed frequencies of 0.26% and 0.13%, respectively.

**Table 1:** Enrichment in amino acid changes related to nucleos(t)ide analog resistance in the pre-treatment quasispecies.

Patient (sample)	Resistance-related aa changes (Mean frequency)	Other aa changes (Mean frequency)	*P*-value
**1 (N = 32 737)**	0.04	0.06	**0.42**
**2 (N = 13 670)**	0.09	0.07	**0.99**
**3 (N = 62 450)**	0.06	0.05	**0.53**
**4 (4A) (N = 32 724)**	0.08	0.03	**0.09**

aa, amino acid; N, number of reads obtained by ultra-deep pyrosequencing.

The mean frequency of amino acid changes related to nucleos(t)ide analog resistance (rtI169T, rtV173L, rtL180M, rtA181T/V, rtT184S/A/I/L/G/C/M, rtA194T, rtA202C/G/I, rtM204V/I [7], rtW153Q [18], rtV191I [19] and rtV207I [20]) obtained by ultra-deep pyrosequencing at pre-treatment was compared with the mean frequency of the remaining amino acid changes.

Patient 1 had detectable percentages of rtA181T and rtM204V/I by UDPS in the baseline quasispecies, but neither was selected during LMV treatment (Table 2). Furthermore, the patient did not experience viral breakthrough (VBK), defined as an increase in serum HBV-DNA of at least 1 log_10_IU/mL compared to the lowest value during treatment, confirmed by real-time PCR [6]. In patients 2 and 3, LMV resistance was associated with predominant selection of rtL180M and rtM204V, despite the fact that higher percentages of rtA181T and/or rtM204I were detected by UDPS at baseline (Table 2). In patient 4, rtA181T and rtM204I were the most prevalent aa substitutions at pre-treatment according to UDPS results; however only rtA181T was detected by LiPA (INNO-LiPA HBV DR v2 assay) and direct sequencing at LMV VBK (Table 2). Furthermore, rtL180M and rtM204V, which were present in frequencies below the mismatch error (0.03%) at pre-treatment, accounted for around 5% of the quasispecies at LMV VBK (sample 4C in Table 3). On linkage analysis, combinations of polymerase variants were not detected in any of the baseline populations, even though more than 60 000 sequences were examined in the sample from patient 3.

**Table 2:** Relationship between frequencies of lamivudine resistance-related amino acid changes detected at pre-treatment and their selection at lamivudine viral breakthrough in the four patients.

Patient (sample)	RT aa changes	Pre-treatment relative frequency by UDPS (%)	Selection at LMV VBK
**1 (N = 32 737)**	**rtL180M**	0	No
	**rtA181T**	0.07	No
	**rtM204I**	0.07	No
	**rtM204V**	0.06	No
**2 (N = 13 670)**	**rtL180M**	0	LiPA & Seq
	**rtA181T**	0.18	No
	**rtM204I**	0.35	No
	**rtM204V**	0.09	LiPA & Seq
**3 (N = 62 450)**	**rtL180M**	0	LiPA & Seq
	**rtA181T**	0.07	No
	**rtM204I**	0.09	No
	**rtM204V**	0.05	LiPA & Seq
**4 (4A) (N = 32 724)**	**rtL180M**	0	LiPA
	**rtA181T**	0.09	LiPA & Seq
	**rtM204I**	0.08	No
	**rtM204V**	0	LiPA

aa, amino acid; LiPA, INNO-LiPA HBV DR v2 assay; LMV, lamivudine, N, number of reads obtained by UDPS; RT, reverse transcriptase; Seq, direct sequencing; UDPS, ultra-deep pyrosequencing; VBK, virologic breakthrough (increase in serum HBV-DNA levels, confirmed by real-time PCR, of at least 1 log_10_IU/mL compared with the lowest value during NA treatment [6]).

Comparison between the relative frequencies of lamivudine-resistant variants detected by UDPS at pre-treatment and their subsequent detection by reverse hybridization (INNO-LiPA HBV DR v2 assay) and/or direct sequencing at lamivudine viral breakthrough in the four patients.

**Table 3:** Frequencies of amino acid substitutions with the highest variability during sequential treatment in each of the five treatment samples.

Amino acid substitutions	4A	4B	4C	4D	4E	*SD*
**L180M**	0	0.01^n^	**5.02**	**0.6**	**99.3**	*48.8*
**M204V**	0.02^n^	0	**5.6**	**0.7**	**99.4**	*48.7*
**S202G**	0	0.02^n^	**0.15**	**0.08**	**80.9**	*40.4*
**V207I**	0.01^n^	0.02^n^	**3.9**	**0.8**	**80.9**	*39.7*
**A181T**	0.09	0.05	**71.7**	**64**	**0.23**	*39.2*
**V173L**	0.05	0.04	**1.4**	**0.13**	**18.2**	*8.9*
**I169T**	0	0	0.04	0	**17.3**	*8.7*
**V191I**	**0.59**	**1.09**	**5.8**	**8.0**	0.02^n^	*3.8*
**M204I**	0.08	0.03	**1**	0.09	0	*0.5*
**T184A**	0.01^n^	0	0.04	0.06	**0.91**	*0.44*
A181S	0.02^n^	0.01^n^	**0.47**	**0.23**	0	*0.22*
A200V	0.02^n^	0.01^n^	0.01^n^	0.04	**0.38**	*0.18*
I162T	0.08	0.06	**0.18**	**0.16**	0	*0.08*
K149N	0.02^n^	0	0.06	**0.17**	0	*0.08*
L157M	**0.19**	**0.14**	**0.19**	**0.16**	0.02^n^	*0.08*
K154Q	0.05	0.01^n^	0.05	**0.18**	0.06	*0.08*
R153W	0.06	0.01^n^	0.05	**0.18**	0.03	*0.07*
V163I	0.10	0.04	**0.17**	**0.14**	0.03	*0.07*
V173M	0.07	0.04	**0.15**	**0.10**	0	*0.06*
K154T	0.01^n^	0.03	**0.10**	**0.13**	0	*0.06*
I169L	0.03	0.03	**0.15**	**0.12**	0.08	*0.06*
R167H	0.06	0.03	0.02^n^	0.04	**0.12**	*0.05*
V207M	**0.13**	0.05	0.04	0.07	**0.12**	*0.04*
G152R	0.08	0.04	0.04	0.03	**0.11**	*0.03*
R153Q	**0.13**	0.06	0.06	**0.12**	0.07	*0.03*
A194T	0.07	0.07	0.05	**0.10**	0.06	*0.02*
**NUMBER OF SEQUENCES**	32724	35662	25289	19740	23249	

SD, standard deviation;

^n^, Frequencies below the mismatch error rate (0.03%).

Frequencies of reverse transcriptase amino acid substitutions with the highest variability during sequential antiviral treatment, according to their standard deviation, in the 5 samples analyzed in patient 4 (4A and 4B correspond to pre-treatment, and 4C, 4D and 4E correspond to completion of lamivudine, adefovir, and entecavir, respectively). Substitutions are arranged in descending order, from the most to the least variable. Frequencies of substitutions ≥0.1% in any sample are shown in bold. Substitutions selected for linkage analysis are shown in bold.

In the overlapping S region (codons s140–s200), 21 substitutions were present at an average frequency of ≥0.1% (data not shown). In the “a determinant”, only substitution sG145R (0.11%), the main HBsAg immune escape variant [26], was present at >0.1%. Among these 21 substitutions, 9 were located in the epitope flanked by codons s156 and s175 (TH-s156/s175), which has been reported to stimulate CD4^+^ T-lymphocytes in subjects receiving the anti-HBV vaccine [27]. Of note, a substantial part of these substitutions (4 out of 9) resulted in stop codons (*) (sW156*, sW163*, sW165* and sW172*), giving rise to proteins with significant aa deletions. In fact, 8 of the 21 substitutions led to a stop codon, which, taken together, represented 0.98% to 2.1% of the 4 baseline viral populations. Four of these 8 stop codons affected RT positions associated with NA resistance (sW172* related to rtA181T, sW182* to rtV191I, sW196* to rtM204I, and sW199* to rtV207I), at frequencies of 0.20% to 0.30%, significantly higher than the artifactual error (0.03%).

### UDPS analysis of conserved amino acid residues

Assessment of the frequencies of synonymous (ds) nt changes (which do not imply an aa change) and non-synonymous (dn) nt changes (which imply an aa change) in the P ORF of the 4 pre-treatment samples (Table S2) revealed 16 conserved positions with a dn ≤0.1%, among which only residue rtL155 showed a dn <0.03% (virtually unchanging) at pre-treatment and during treatment (Figure 2). In the essential YMDD catalytic motif, only residue rtY203 was included within the 16 positions, whereas rtD205 and rtD206 showed relatively high variability, an unexpected finding considering their essential role in polymerase catalytic activity [28]. In the S ORF, ten positions showed a dn ≤0.1% (Table S2). Three of them were located in the “a determinant”: sP142 and sN146, associated with viral infectivity [29], and sC147, a cysteine critical for secretion of HBV particles [30]. Other potentially essential positions included in the fragment analyzed, such as sS174, proposed as a “hot spot” for Surface/Core protein-to-protein interactions based on a predicted 3Dmodel of the surface protein [30], were not among the most highly conserved.

**Figure 3:**
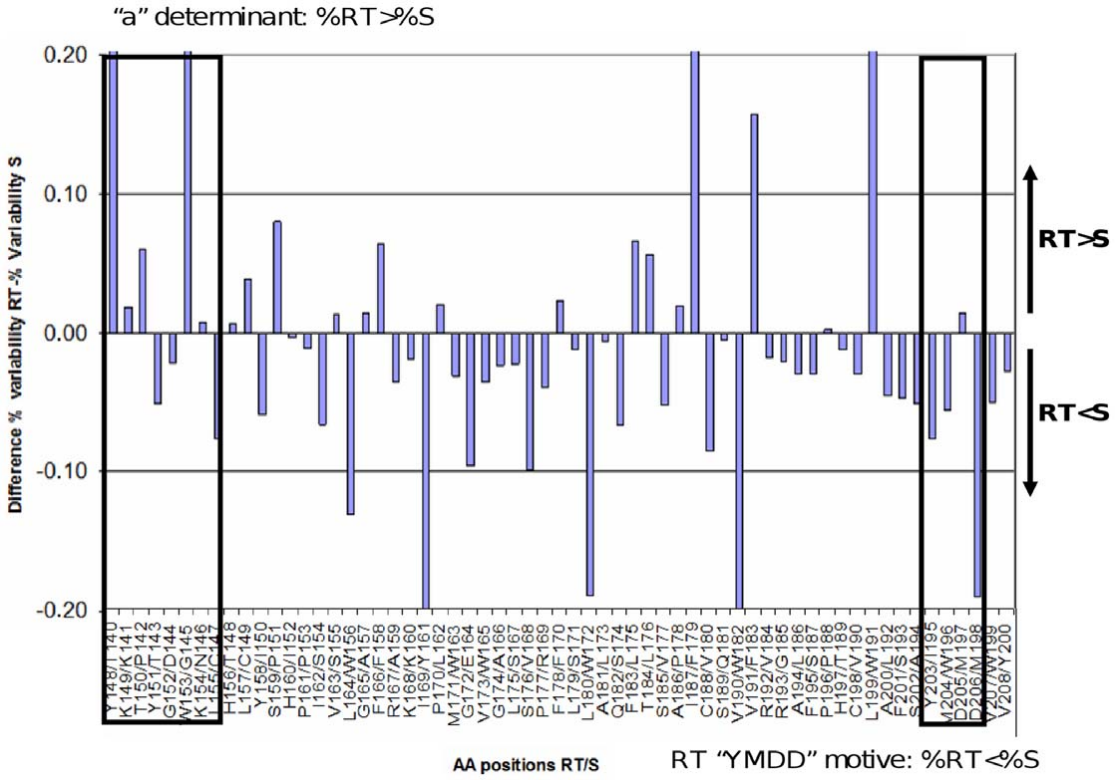
Differences in variability throughout the fragment of reverse transcriptase and overlapping surface region analyzed. In the pre-treatment samples (1 to 4A), each bar represents the difference between the average frequency of amino acid substitutions in each reverse transcriptase (RT) position and its overlapped amino acid in the surface (S) region, assuming that any substitution in the first nucleotide of an RT codon affects the third nucleotide of the overlapping S codon. Bars with positive values indicate positions with a higher tolerance for amino acid substitutions in RT than in S, whereas the opposite is indicated by bars with negative values.

To evaluate the relative variability of the P and S ORFs along the region analyzed, we subtracted the percentage of variability of the S region from the percentage of variability of RT region. This calculation was performed taking into account that any change in the first nt of the P codons affects the third nt of the overlapping S codon (P_1_/S_3_), while the second and third nts of P affect the first and second nts of the overlapping S codon (P_2–3_/S_1–2_). In both calculations (P_1_/S_3_ and P_2–3_/S_1–2_), S was more variable than P. Moreover, P_1_/S_3_ showed that in the C-terminal end of the UDPS-analyzed fragment (rtR192-rtV208 corresponding to sV184-sY200), which includes the YMDD catalytic domain, the P ORF was significantly better conserved than the S ORF, whereas in the N-terminal end (rtY148-rtY158 corresponding to sT140-sI150), which includes several residues important for viral infectivity such as those of the “a determinant” [29], [31], the S protein was more highly conserved (Figure 3).

### Longitudinal UDPS analysis of serum samples from a patient with multidrug resistance during sequential therapy

A total of 136 708 validated reads corresponding to 5 serial samples from patient 4, (4A and 4B at pre-treatment and 4C to 4E in sequential treatment) were analyzed. The most variable RT aa substitutions in the 5 samples, sorted by decreasing SD value, are shown in Table 3. The 10 most variable substitutions (highest SD value), rtL180M, rtM204V, rtS202G, rtV207I, rtA181T, rtV173L, rtI169T, rtV191I, rtM204I and rtT184A, are known to be associated with NA resistance. Interestingly, immediately after these well known NA-resistant variants, in positions 11 and 12, we found the rare variants rtA181S and rtA200V, which are associated with ADV and L-nucleoside analogue (LMV and LdT) resistance, respectively [32]. In contrast, rtR153Q and rtA194T, also associated with NA resistance, were less variable (positions 25 and 26 in Table 3).

**Figure 4:**
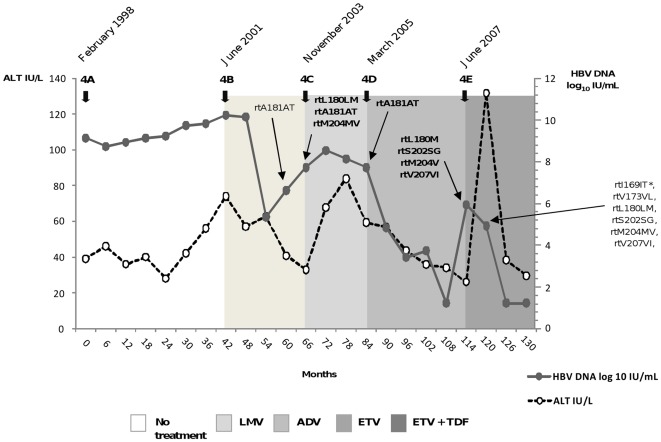
Longitudinal virologic and biochemical outcome of patient 4. Thick arrows indicate the sampling time and thin arrows specify mutations detected by reverse hybridization (INNO-LiPA HBV DR v2 assay) and/or direct sequencing, which are shown in bold in samples analyzed by UDPS. * This variant was not consistently observed by Sanger sequencing.

Changes occurring in the HBV quasispecies of the RT region studied over a 42-month period in the absence of treatment were assessed by comparative UDPS analysis of samples 4A and 4B. The consensus sequence was identical in both samples and the variants showed low comparative changes in frequency, except for rtV191I, which increased from 0.6% (4A) to 1.1% (4B) (Table 3). At the time of LMV VBK (sample 4C), percentages of rtA181T (71.7%), rtM204V (5.6%), and rtL180M (5%) notably increased relative to baseline samples, as did other LMV resistance substitutions, particularly rtV191I (5.8%), rtV207I (3.9%), and rtV173L (1.4%), although to a lesser extent. The ETV-associated substitution rtS202G also increased slightly (0.15%) (Table 3). At the end of ADV (sample 4D), rtA181T, which was detected by LiPA, remained as the major variant (64%) (Table 3 and Figure 4), whereas rtV191I (8%) continued increasing. Moreover, the number of minor substitutions with frequencies ≥0.1% increased with respect to LMV VBK (Table 3). At ETV VBK (sample 4E), two variants previously detected by LiPA at LMV VBK were strongly selected, rtL180M (99.3%) and rtM204V (99.4%) (Table 3 and Figure 4). In addition, other NA-resistant variants related with resistance to ETV and detected by Sanger sequencing also increased significantly at ETV VBK: rtV173L (18.2%), which was detectable by LiPA, rtS202G (80.9%) and rtI169T (17.3%), inconsistently observed by Sanger [22] (Table 3 and Figure 4). We also observed an increase in other NA-resistant variants that have not been previously related to ETV resistance, such as rtV207I (80.9%), detected by Sanger sequencing, and the rare substitution, rtA200V (0.38%) (Table 3 and Figure 4).

In the overlapping S ORF, 51 aa substitutions were present at frequencies ≥0.1% in at least one of the 4A to 4E samples (data not shown). The most variable positions were those that overlapped RT residues associated with NA resistance, particularly sI195M, related to rtM204V, present in 5.6% and 99% of sequences at LMV VBK and ETV VBK, respectively. As observed in the baseline quasispecies, a significant portion of the 20 most frequent variants (highest average substitution frequencies in the 5 samples) led to stop codons (sW156*, sW172*, sW182*, sW191*, sW196* and sW199*), especially at LMV VBK and at the end of ADV, mainly due to mutation sW172*, related to rtA181T, (71.7% and 64%, respectively) and sW182*, related to rtV191I, (5.8% and 8%, respectively). The total frequency of genomes carrying a stop codon was 80%, 74%, and 4.4% at LMV, ADV, and ETV VBK, respectively. These 20 variants did not include the immune escape variant sG145R, detected in relatively high frequencies at pre-treatment, but otherwise included the variants sS167L, sW172C, and sW172*, located at the minimal recognized sequence (positions s165 to s172) of the TH-s156/s175 epitope [27]; among these, sS167L showed a continuous percentage increase from pre-treatment to ETV-VBK (sample 4A: 0.2%, 4B: 0.7%, 4C: 1.1%, 4D: 1.3%, 4E: 4%).

For each sample, nt and aa divergences were assessed as the percentage of sequences different from the master sequence in the first baseline sample (sample 4A), and as the percentage of sequences different from each sample's master sequence. In each quasispecies, nt divergence was always higher than aa divergence in both analyses. Nucleotide divergence in relation to the baseline master sequence was similar in both pretreatment samples (9.72% and 8.23%), but significantly increased during antiviral treatments (82.5% after LMV, 70.84% after ADV and 99.69% after ETV). Amino acid divergence was higher in the S ORF than in P in both pre-treatment samples using both calculations, but under antiviral treatments, aa divergence in the S ORF continued higher than in the P ORF when related to the master sequence of each sample, while they tended to equalize in relation to the baseline master sequence (Table 4).

**Table 4:** Nucleotide and amino acid divergences in the S and P ORFs in each sample from patient 4.

		4A	4B	4C	4D	4E
	**NT Master**	9.72	8.23	82.50	70.84	99.69
**Baseline Master**	**P Master**	7.60	5.57	82.03	69.70	99.69
	**S Master**	9.64	7.34	80.59	70.37	99.67
	**NT Master**	9.72	8.23	41.63	51.33	35.53
**Sample Master**	**P Master**	7.60	5.57	39.65	48.17	31.01
	**S Master**	9.64	7.34	41.05	50.56	33.95

NT Master, nucleotide master sequence; P Master, amino acid master sequence in polymerase (P) ORF; S Master, amino acid master sequence in surface (S) ORF.

Nucleotide and amino acid divergences in samples from patient 4 (4A and 4B correspond to pre-treatment, and 4C, 4D and 4E correspond to completion of lamivudine, adefovir, and entecavir, respectively). In each sample, divergences were calculated as the percentage of all sequences different from the master of the first baseline sample processed (sample 4A) (Baseline Master), and the percentage of all sequences different from the master of the sample where they were obtained (Sample Master).

### UDPS linkage analysis to establish the combination of the most variable residues in the same sequence

**Figure 5:**
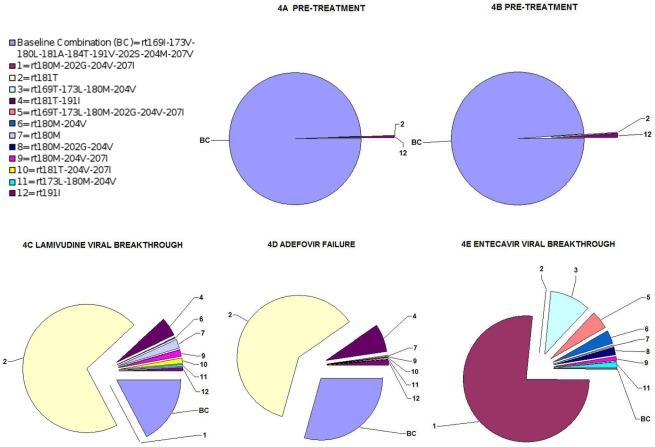
Changes in percentages of reverse transcriptase variants during follow-up of patient 4. Frequencies of the most variable variants in the 5 samples from patient 4 (ie, those detected in frequencies ≥1% by linkage analysis in any of the samples) are shown in a pie chart for each sample. Each variant has been numbered as sorted in Table 5 (by decreasing SD of the frequency), except for the variant with no mutations in any of the positions included in the analysis. This variant, which was the most variable of all, is designated *baseline combination*.

The 10 most variable RT substitutions observed during sequential NA treatment, blindly selected after SD sorting and all associated with NA resistance (rtL180M, rtM204V, rtS202G, rtV207I, rtA181T, rtL173V, rtI169T, rtV191I, rtM204I, and rtT184A) (Table 3), were included in the linkage analysis. Among all their possible combinations, the sequence with no mutations in these positions (baseline combination: BC) showed the highest variability during NA treatment. This BC sequence was largely dominant at pre-treatment (98.5%), but greatly decreased at VBK during sequential NA treatment, especially at ETV VBK (0.3%) (Table 5 and Figure 5).

**Table 5:** Linkage analysis of the most variable amino acid substitutions during sequential treatment.

Combined variants	4A	4B	4C	4D	4E	*SD*
**169I-173V-180L-181A-184T-191V-202S-204M-207V (BC)**	**98.54**	**98.51**	**19.60**	**33.80**	0.30	*43.5*
**180M-202G-204V-207I**	0.00	0.00	0.10	0.01^n^	**72.36**	*36.2*
**181T**	0.09	0.05	**64.08**	**56.45**	0.10	*34.9*
**169T-173L-180M-204V**	0.00	0.00	0.00	0.00	**10.00**	*5.0*
**181T-191I**	0.00	0.00	**4.39**	**6.57**	0.01^n^	*3.3*
**169T-173L-180M-202G-204V-207I**	0.00	0.00	0.01^n^	0.00	**4.59**	*2.3*
**180M-204V**	0.00	0.00	0.28	0.01^n^	**3.20**	*1.6*
**180M**	0.00	0.00	**1.94**	0.27	0.11	*0.9*
**180M-202G-204V**	0.00	0.00	0.00	0.00	**1.82**	*0.9*
**180M-204V-207I**	0.00	0.00	**1.38**	0.22	**1.19**	*0.7*
**181T-204V-207I**	0.00	0.00	**1.24**	0.18	0.00	*0.6*
**173L-180M-204V**	0.00	0.00	0.59	0.05	**1.26**	*0.6*
**191I**	0.59	**1.08**	0.78	**1.32**	0.00	*0.6*
**173L-180M-184A-204V**	0.00	0.00	0.00	0.00	0.89	*0.4*
**169T-180M-202G-204V-207I**	0.00	0.00	0.00	0.00	0.76	*0.4*
**169T-173L-180M-204V-207I**	0.00	0.00	0.00	0.00	0.74	*0.4*
**181T-204V**	0.00	0.00	0.75	0.07	0.00	*0.4*
**173L-180M-202G-204V-207I**	0.00	0.00	0.00	0.00	0.55	*0.3*
**204V-207I**	0.00	0.00	0.57	0.11	0.00	*0.3*
**173L-180M**	0.00	0.00	0.55	0.06	0.00	*0.3*
**169T-180M-184A-202G-204V-207I**	0.00	0.00	0.00	0.00	0.53	*0.3*
**204V**	0.02^n^	0.00	0.47	0.06	0.00	*0.2*
**204I**	0.08	0.03	0.43	0.02^n^	0.00	*0.2*
**169T-173L-180M-184A-204V**	0.00	0.00	0.00	0.00	0.40	*0.2*
**173L-180M-184A-202G-204V-207I**	0.00	0.00	0.00	0.00	0.36	*0.2*
**181T-207I**	0.00	0.00	0.26	0.21	0.00	*0.1*
**169T-180M-204V**	0.00	0.00	0.00	0.00	0.26	*0.1*
**181T-204I**	0.00	0.00	0.26	0.07	0.00	*0.1*
**169T-173L-180M-202G-204V**	0.00	0.00	0.00	0.00	0.18	*0.1*
**180M-184A-204V**	0.00	0.00	0.00	0.00	0.18	*0.1*
**169T-173L-180M-184A-202G-204V-207I**	0.00	0.00	0.00	0.00	0.17	*0.1*
**204I-207I**	0.00	0.00	0.15	0.00	0.00	*0.1*
**207I**	0.01^n^	0.02^n^	0.13	0.08	0.00	*0.1*
**173L-181T**	0.00	0.00	0.12	0.02^n^	0.00	*0.1*
**173L**	0.05	0.03	0.06	0.01^n^	0.00	*0.0*
**184A**	0.05	0.01^n^	0.05	0.02^n^	0.00	*0.0*
**202G**	0.00	0.02^n^	0.01^n^	0.04	0.00	*0.0*
**NUMBER OF SEQUENCES**	32724	35662	25289	19740	23249	

SD, standard deviation;

^n^,Frequencies below the mismatch error rate (0.03%); BC, Baseline combination (the master combination of positions where substitutions with the highest variation, shown in bold in Table 3, were located).

Frequencies of combinations (linkage) of the 10 amino acid substitutions with the highest standard deviation of frequencies in the 5 sequential treatment samples from patient 4 (4A and 4B correspond to pre-treatment and 4C, 4D and 4E correspond to completion of lamivudine, adefovir, and entecavir, respectively). The variant combinations are presented in decreasing order (from most to least variable). Frequencies of combinations ≥1% are shown in bold.

Linkage analysis yielded two main findings: First, the 10 most variable aa substitutions were mainly present in variant combinations, the only exception being rtA181T, which was the single substitution in 64.1% of sequences at LMV VBK and 56.5% at the end of ADV (Table 5). This substitution was also found in combination with rtV191I in a significant percentage of variants (4.4% at LMV VBK and 6.6% at ADV end) (Table 5). Second, at ETV VBK, rtI169T and/or rtS202G, which are typically associated with ETV resistance, always appeared linked to the LMV resistance signature (rtL180M-rtM204V), in keeping with previous reports [22] (Table 5), whereas rtV207I, which has not been previously associated with ETV resistance, unexpectedly appeared in most of the highly frequent combinations and always together with other variants, especially rtM204V (Table 5). In order to confirm whether the association of rtV207I and rtM204V was specific to this patient or a more general phenomenon, results from a first-generation LiPA strip (which included NA resistance-related substitutions in codons rt204 and rt207 [33]) of 50 patients who had failed LMV treatment were retrospectively checked. Thirty-nine percent of them showed rtV207I and rtM204V/I (data not shown). Three patients carrying rtM204V and rtV207I had been subsequently treated with ETV, and two of them had rapid VBK after an initial ETV response.

The predominant presence and complexity of the combined aa substitutions was particularly evident at ETV VBK, in which the main variant combination was rt180M-202G-204V-207I (72.4%), followed by rt169T-173L-180M-204V (10%) and rt169T-173L-180M-202G-204V-207I (4.6%) (Table 5 and Figure 5). To support the reliability of the frequencies of combinations detected, the 4E sample was analyzed by molecular cloning. In an analysis of 23 clones, the rt180M-202G-204V-207I combination was also the most prevalent (65% of clones), followed by rt169T-173L-180M-204V (22%), and rt180M-204V (13%). UDPS showed that many of the most prevalent combinations detected at ETV VBK were present in ≥0.1% at LMV VBK, such as: rt180M-202G-204V-207I (0.1%), rt180M-204V (0.3%), rt180M-204V-207I (1.4%), and rt173L-180M-204V (0.6%) (Table 5 and Figure 5). Among them, combined variants carrying only LMV resistant mutations, such as rtV173L-rtL180M-rtM204V and rtL180M-rtM204V, increased in frequency from LMV to ETV VBK (from 0.6% to 1.3% and from 0.3% to 3.2%, respectively) (Table 5 and Figure 5).

## Discussion

Massive UDPS analysis enabled sensitive baseline detection of minor viral populations associated with NA resistance, as has been reported [34], and identification of highly variable and conserved residues. In addition, UDPS proved to be a powerful technique for quantitative study of the dynamics of HBV populations resulting from the multiple evolutionary pressures of sequential NA therapy. The capability to clonally analyze thousands of sequences disclosed combinations of aa substitutions occurring in the same genome during antiviral treatment.

Identification of mutations in extremely low percentages with an acceptable degree of confidence is limited by the number of independent template molecules obtained from the sample analyzed, the coverage or number of reads obtained per base, and the number of artifactual errors generated during PCR amplification and pyrosequencing [13]. For these reasons, all the samples selected for the current study carried a high HBV viral load (>10^5^ IU/mL), and the high-fidelity DNA polymerase Pfu turbo linked to a Poisson-based computational algorithm [13] was used to bypass the artifactual errors. With this approach, variants comprising as little as 0.03% of the HBV quasispecies could be detected. UDPS analysis showed that aa changes known to be associated with NA resistance above this detection limit (rtA181T, rtV191I, rtA194T and rtM204I) were present in low percentages (ranging from 0.07 to 0.59) in the baseline HBV quasispecies, probably representing a background due to the natural dynamics of the viral quasispecies. Moreover, variant combinations were not detected in the baseline quasispecies. These results suggest that if the resistance changes were present (which seems likely because of their strong selection in the longitudinally analyzed patient 4), they would be in percentages below the detection limit. Therefore, higher sensitivity than is reported here seems to be required for detecting combined variants at baseline. In the sequentially treated patient, the relative frequency of NA-resistant substitutions in the baseline quasispecies did not seem to be predictive of subsequent LMV treatment outcome or RT variants selected at VBK.

The high sensitivity of UDPS also made possible identification of conserved residues. The residue rtL155 was found to be the most highly conserved at both pre-treatment and throughout sequential treatment, a previously unreported finding. According to a structural analysis based on a 3Dmodel of HBV RT [35], this leucine residue is located at the external surface of RT (Figure S1), and has high hydrophobicity, features that suggest a role in protein-to-protein interactions. The low overall frequency of aa substitutions in rtL155 was even lower than that observed in residues deemed essential for viral polymerase function, such as rtY203, rtD205 and rtD206 from the conserved YMDD catalytic motif [28], [36]. Considering the essentiality of rtD205 and rtD206, which are part of the catalytically essential aspartic acid triad of HBV RT [28], a higher than expected variability was found (>0.1% in one case). In this sense, it has been reported that substitutions in rtD205 result in replication-defective HBV variants that can be trans-complemented in vitro by wild-type polymerases [37]–[39]. This mechanism may explain the finding of genomes with aa substitutions in rtD205, which may replicate through trans-complementation with a helper wild-type HBV polymerase in the same hepatocyte.

The HBV genome has an extremely overlapping structure [4]. With UDPS, we were also able to study epitopic regions of the S ORF that overlap the RT region. In the baseline viral populations, the S ORF showed significant percentages of substitutions that lead to a stop codon (1%–2%). These mainly overlapped RT positions related to NA resistance variants, and some of them delete important envelope residues involved in viral infectivity and/or possible interactions with core proteins [29], [30]. The envelope stop codon variant sW172*, which is related to the major NA-resistant variant rtA181T, became the most highly represented following LMV and ADV treatments, yielding a major viral population that is defective in secretion of viral particles, as reported by Warner et al. [23]. This strongly suggests that secretion of genomes harboring the sW172* substitution would be enabled by trans-complementation with a functional S protein from other HBV genomes occurring in the same quasispecies, as previously suggested [24]. Trans-complementation of envelope-defective variants may be favored by the huge excess of HBsAg production during chronic infection (1000- to 10 000-fold excess in relation to complete viral particles) [40]. Thus, envelope-competent genomes might produce enough HBsAg for their own envelops and those of the defective genomes, even as minor viral populations. These findings suggest that the large excess of HBsAg may have evolved to offset the presence of envelope stop codons.

Regarding the relative variability of the P and S ORFs, we found that the N-terminal region (rtY148-rtY158 corresponding to sT140-sI150) included in the “a determinant”, which is the main target for anti-HBs neutralizing antibody [41], [42], was more conserved in the S than in the P ORF. This may be explained by the close relationship of this epitope with infectivity [31]. Moreover, the only variant found in proportions above 0.1% was the well-known immune escape substitution sG145R [26], which modifies the antigenicity of the “a determinant”, while viral particles remain infective [29]. Interestingly, despite the relevance and high degree of conservation of the “a determinant”, RT residue rtL155 was more highly conserved than its overlapped amino acids in the S ORF, sN146 and sC147, both essential for the structure and function of this determinant [29], [30]. Contrary to what was observed for the N-terminal region, in the C-terminal (rtR192-rtV208 corresponding to sV184-sY200), where the essential YMDD motif of RT is located, the P ORF was more conserved than the S ORF. These observations support the notion that although the polymerase and surface proteins share the same nt sequence, they evolve independently to preserve their essential functions, as reported by van Hemert et al. [30].

In the longitudinal study of the patient sequentially treated with LMV, ADV, and ETV monotherapies, the blind SD-based algorithm ranked the 7 NA resistance-related aa substitutions previously detected by routine analysis (rtL180M, rtM204V, rtS202G, rtV207I, rtA181T, rtV173L and rtI169T) as the most variable, and 5 additional NA resistance substitutions (rtV191I, rtM204I, rtT184A, rtA181S and rtA200V) immediately after them. These findings confirm the utility of this methodology as a “scanning tool” to detect, without any previous assumptions, the most relevant substitutions associated with MDR, even some that would not be found unless specifically checked. The use of UDPS as a scanning tool linked to its ability to quantify the frequencies of HBV variants could provide a measure of their relative sensitivity to different NA therapies. In this sense, the rtA181T variant, which strongly increased from pre-treatment to LMV and slightly decreased during ADV therapy, appeared to be more resistant to LMV than to ADV, in agreement with its previous phenotypic characterization [24]. With this approach, the sensitivity to different NAs of the less common resistant variants (rtV191I, rtA181S, and rtA200V) found among the most variable ones can also be studied. In this particular case, percentages of rtV191I increased during LMV and ADV, mainly in combination with the major variant rtA181T, suggesting a compensatory role of rtV191I to restore its replicative fitness. During ETV, percentages of both rtA181T and rtV191I dramatically decreased, indicating sensitivity to this drug. The variation in percentages of rtA181S followed a pattern similar to that of rtA181T, but with a less intense effect (moderately increased after LMV VBK, maintained during ADV, and undetectable after ETV treatment), therefore, position rt181 had a major role in resistance to multiple NAs in the longitudinally followed patient. Moreover, rtA181S is linked to the sW172C substitution in the minimal recognized sequence of the surface epitope TH-s156/s175 [27]; hence, it is likely to provide immune escape. In the case of the rtA200V substitution, although previously associated with resistance to LMV and LdT [32], in this longitudinal study it was only found significantly increased at ETV VBK, suggesting some “decreased sensitivity” to ETV. In the overlapping S ORF, quantitative UDPS analysis was applied to study the sensitivity of the HBV variants to immune pressure. In this sense, the increase in percentages of NA-resistant rtV191I in the absence of treatment concurs with its reported link to humoral immune response escape by an association with the surface stop codon sW182* [19], recently related to liver disease progression [43]. In addition, the sS167L variant, associated with a silent RT substitution in rtL175 (CTC to CTT), showed a continuous percentage increase in the absence of treatment and during follow-up. sS167 is located in the minimal recognized sequence of the TH-s156/s175 epitope, and changes in this residue have been reported in patients simultaneously showing HBsAg and anti-HBs antibodies [42]. Therefore, it would seem to be subjected to immune selection rather than treatment selection. Hence, the findings in the RT and S ORFs observed in the sequentially treated patient suggest that UDPS may have value as a “phenotype-like” assay to assess the influence of any type of selective pressure on the composition of viral quasispecies.

Quantitative UDPS analysis is not only useful to analyze particular positions of the HBV genome, but also to examine changes in the variability of the quasispecies by quantifying the global nt and aa divergences of its sequences in relation to the master sequence. In the longitudinally studied case, the high increase in nt divergence over several antiviral treatments reflects adaptation of the viral quasispecies by selection of antiviral-resistant variants. The higher divergence in nt sequences in comparison to aa sequences is likely related to genetic code degeneration. Furthermore, the higher aa divergence in S than in P ORFs observed when related to the master sequence of each sample may be evidence of immune pressure over envelope proteins, which are more prone to globally evolve than polymerase due to functional constrictions, especially in the absence of antiviral treatment. However, P and S divergences tended to equalize when they were analyzed in relation to the baseline master sequence (sample 4A). This pattern may reflect an evolutive pressure over P ORF positions affecting in parallel the corresponding overlapped S ORF positions.

The superiority of GS-FLX technology for clonally sequencing relatively long single DNA molecules as compared to other massive sequencing platforms [44] enabled study of particular combinations of two or more aa substitutions in the same genome by linkage analysis. To facilitate computation and interpretation of these analyses, we focused on the 10 most variable substitutions, assuming that they would be the most relevant to study HBV adaptation to the pressure of sequential NA treatment. Using this approach, we found that in our MDR patient, with the single exception of rtA181T, all these substitutions were mainly found in combinations in all the samples studied. The substitution patterns were particularly complex at ETV VBK, with rtL180M-S202G-M204V-V207I being the main combination. This pattern was confirmed by molecular cloning, and had already been detected as a minor population at LMV VBK, followed by rt169T-173L-180M-204V and rt169T-173L-180M-202G-204V-207I (6 mutations in a single genome). In this particular case, the frequency of the LMV resistance signature (rtL180M-M204V) alone showed a considerable increase at ETV VBK relative to its frequency after LMV, as also occurred with the more complex variant rtV173L-L180M-M204V. This suggests that LMV-resistant mutants have slightly reduced sensitivity to ETV, as reported by phenotypic assays [22], [45], [46]. Unexpectedly, the rtV207I variant, which to date has only been associated with low sensitivity to LMV and resistance to famciclovir [20], was present in the most prevalent combined variants at ETV VBK (81% of sequences) and was also significantly increased at LMV VBK. This variant was mainly selected in combination with rtM204V, a fact that strongly suggests a compensatory role to restore the replicative efficiency of complex HBV variants carrying rtM204V, likely to compromise the success of ETV. Although these observations were obtained in a single case, this hypothesis is supported by our retrospective review of LiPA results. However, in contrast to this hypothesis, the sensitivity of the LMV-resistant signature to ETV did not change when the signature was combined with rtV207I in a previous phenotypic study [46]. Therefore, additional phenotypic analysis should be performed taking into account the complexity of the variants detected here by linkage analysis.

Our study has some limitations. Only a small number of serum samples could be analyzed because of the high cost of the technique and complexity of the computational analysis. Moreover, because only a single MDR patient was longitudinally analyzed, the results obtained regarding changes in the viral quasispecies under the effect of antiviral treatments require corroboration by further studies examining additional patients. In addition, due to the 250-bp-length limitation of the standard GS-FLX chemistry, the relevant NA-resistant substitutions, rtI233V and rtN236T linked to ADV treatment failure, and rtM250I/V linked to ETV failure [7], located outside the B and C HBV RT functional domains, were excluded from the fragment analyzed. However, this last limitation was not considered relevant because we only selected patients who did not show NA resistant substitutions outside the region analyzed, as assessed by LiPA and/or direct sequencing during follow-up.

To summarize, UDPS detected minor variants comprising less than 0.1% of the HBV viral quasispecies. Nonetheless, the information provided did not enable prediction of which resistant aa substitutions would be selected during treatment. Additional studies are needed to determine at what frequency HBV variants become clinically relevant. However, the high sensitivity of this technology has resulted in some unexpected findings: first, the high degree of conservation of residue rtL155 and a significant percentage of defective genomes at baseline (with variations in essential residues of the RT active site or stop codons in the S ORF) that became the predominant population after LMV and ADV treatments. These results suggest that the HBV quasispecies has an active trans-complementation mechanism enabled by coinfection of cells with multiple variants. Second, as tested in one sequentially treated patient, assessing and ranking the variability of aa substitutions through sequential treatment using a “blinded” algorithmic method driven by an objective variability measure (SD) of their frequencies highlighted the most important substitutions occurring during this period, with no need for previous knowledge about HBV variants and their resistance to antiviral treatments. Therefore, this method can potentially act as a “scanning tool” to detect new resistant variants in viral quasispecies, and indicates a role as a “phenotype-like” method that provides information on the relative susceptibility of these variants to any type of selective pressure (eg, antiviral therapies or immune pressure). Quantitative UDPS analysis was also useful to analyze the global variability of the HBV quasispecies and its evolution, by quantifying the nt and aa divergences of its sequences. Lastly, the partial picture of reality provided by UDPS analysis of individual substitutions is significantly improved by linkage analysis, which allows detection and quantification of variant combinations, which seem to be the most common cause of resistance in anti-HBV therapy in our sequentially treated patient. In conclusion, UDPS offers significant advantages for the study of viral quasispecies, although currently its potential is mainly limited by its high cost. As new applications for this technology are developed, it is likely that the cost will significantly decrease.

## Methods

### Ethics statement

The study protocol was approved by the Ethics Committee for Clinical Investigation of University Hospital Vall d'Hebron. Informed written consent from participating patients was obtained, according to the Declaration of Helsinki.

### Patients and samples

Serum samples from 4 CHB patients were included in the study. To obtain comparable results, patients were selected according the following criteria: first, all were hepatitis B e antigen (HBeAg)-positive and all had HBV genotype A, subgenotype A2, assessed by direct sequencing and alignment as previously reported [35]; second, they were initially treated with LMV and no resistance variants were found at baseline, as assessed by LiPA (INNO-LiPA HBV DR v2 assay, Innogenetics, Ghent, Belgium) or direct sequencing; and third, HBV NA-resistant variants outside the B and C domains of the RT were not selected in any case after NA treatment. The baseline characteristics and outcome of initial LMV treatment are summarized in Table 6. Patient 1 was only treated with LMV and showed virologic response (undetectable HBV-DNA levels by real-time PCR within 48 weeks of therapy [6]) and HBeAg seroconversion (loss of HBeAg with detection of anti-HBe antibodies) (Table 6). In contrast, patients 2, 3 and 4 experienced VBK with selection of resistant variants (Table 6). In patients 2 and 3, ADV was added after LMV VBK, and both showed virologic response with undetectable HBV-DNA levels after stopping LMV. In addition, patient 3 lost HBeAg after 3 years of ADV+LMV and additionally lost HBsAg in the following 3 years with ADV monotherapy. In patient 4, LMV failure was associated with emergence of the rtA181T variant (Figure 4). The patient, who was subsequently treated with ADV and ETV monotherapies, showed no response to ADV and had VBK after ETV with selection of resistant variants (Figure 4). TDF was then added to ETV, and the patient achieved virologic response without HBeAg loss up to the time this study was performed (Figure 4).

**Table 6:** Baseline characteristics of the patients included and outcome during LMV treatment.

BASELINE CHARACTERISTICS					LAMIVUDINE TREATMENT		
*Patient*	*Age*	*Gender*	*HBV-DNA (log_10_IU/mL)*	*ALT (IU/L)*	*Duration (months)*	*Type of Response*	*Resistant variants at VBK*
1	70	F	6.54	112	28	HBeAg seroconversion and virologic response	None
2	52	M	6.08	53	51	Partial virologic response (2.2 logΔ) and VBK (7.7 log*)	rtM204V+rtL180M
3	54	M	7.08	73	39	Partial virologic response (4.2 logΔ) and VBK (7.3 log*)	rtM204V+rtL180M
4	32	M	8.44	39	19	Partial virologic response (2.6 logΔ) and VBK (7.2 log*)	rtM204V+rtL180M+rtA181T

F, female; M, male; HBeAg, hepatitis B e antigen; log, HBV-DNA (log_10_IU/mL);

Δ , HBV-DNA decrease 6 months after beginning lamivudine treatment relative to baseline;

* , HBV-DNA levels at virologic breakthrough, which determined the end of lamivudine treatment and occurred at the end of the period indicated in the Duration column.

HBV-DNA was quantified using real-time PCR (Cobas Ampliprep-Taqman, Roche; sensitivity 20 IU/mL), and resistant variants were determined in the sample obtained at viral breakthrough by LiPA (INNO-LiPA HBV DR v2 assay) and/or direct sequencing. The response to lamivudine treatment was defined as:

- HBeAg seroconversion: Loss of hepatitis B e antigen with detection of anti-HBe antibodies.

- Virologic response: undetectable HBV-DNA levels by real-time PCR within 48 weeks (12 months) of therapy [6].

- Partial virologic response: decrease in HBV DNA of more than 1 log_10_IU/mL, but detectable HBV DNA by real-time PCR, assessed at 24 weeks (6 months) of treatment, for lamivudine [6].

- VBK: virologic breakthrough - increase in serum HBV-DNA levels of at least 1 log_10_IU/mL compared to the lowest value during NA treatment, confirmed by real-time PCR [6].

Eight serum samples were selected for UDPS analysis: 1 sample obtained before starting LMV therapy in patients 1, 2 and 3; and 5 consecutive samples from the sequentially treated patient 4, among which 2 samples (4A and 4B) were obtained before starting LMV in a 42-month period without treatment, and 1 sample each was obtained at the end of LMV (4C), ADV (4D) and ETV (4E) treatment (Figure 4).

### HBV genome region analyzed by UDPS

To perform linkage analysis of the major NA-resistance-related aa substitutions in the RT region, a single fragment clustering these substitutions was analyzed. At selection of this fragment, the idea was to include the HBV RT B and C domains, which comprised all NA-resistant mutations previously detected by LiPA or direct sequencing at VBK in each patient (Table 6 and Figure 4) (third patient's inclusion criteria). In addition, to assess the changes in the most important epitopes of the overlapped S ORF, the fragment included the largest part possible of the C-terminal region of the immunodominant epitope “a determinant”, which had the main immunotherapy escape mutation sG145R, associated with the rtW153Q NA compensatory variant [18]. Finally, we had to restrict the length of the fragment to 250 bp as required by the standard GS-FLX technology, the only one available when the study was designed. These criteria were fulfilled by a fragment of 224 bp, in which 180 bp were analyzed after excluding the known sequences of the primers. The fragment selected covers an HBV P-S overlapping sequence spanning codons rt148 to rt208 in the P ORF and codons s140 to s200 in the S ORF (Figure 1). NA-resistance-related aa substitutions outside the B and C domains, such as rtI233V, rtN236T and rtM250I/V, were not considered relevant for the aims of the study, since they had not been detected during follow-up in any of the patients studied.

### PCR amplification of the region analyzed

A 746-bp fragment (nt 246 to 992) of HBV-DNA including the region for UDPS analysis was amplified by PCR using primers F2 and R2 [47]. A second PCR run amplified the specific UDPS fragment (nt 551 to 775) with fusion primers HBVRTfw: 5′-*GCCTCCCTCGCGCCATCAG*ATGTTTCCCTCATGTTGCTG-3′ and HBVRTrv 5′-*GCCTTGCCAGCCCGCTCAG*CTGTACAGACTTGGCCCCCA-3′ (the 5′ adaptor sequences that act as binding sites for the emulsion PCR and pyrosequencing reaction of the UDPS system, both ending with the TCAG barcode, are shown in italics). This second PCR run generated a 262-bp amplicon (Figure 1). To minimize misincorporation errors, the high-fidelity Pfu Turbo DNA polymerase (Stratagene, Agilent Technologies, La Jolla, USA) was used in both PCR runs [13], [48]. The length and quality of the amplicons obtained were verified by automated chip-based microcapillary electrophoresis (Agilent 2100 Bioanalyzer instrument, Agilent Technologies, Santa Clara, USA) and quantified (Quant-it PicoGreen dsDNA fluorescent Assay Kit, Invitrogen, Carlsbad, USA). Ten µL of each amplicon, with a concentration ≥0.5 ng/µL (≥10^7^ copies/µL) underwent UDPS with the GS-FLX platform, according to the manufacturer's protocol.

The 746-bp amplicon obtained in the first PCR round from sample 4E was directly cloned, as previously described [49].

### Quantification of artifactual errors

The main error source for the detection of single-base changes when deep-sequencing an amplicon seems to be polymerase mismatch errors [50]. To quantify these errors as has been done in previous studies [13], [16], a DNA clone with a known sequence was UDPS-analyzed in triplicate and sequenced by the conventional Sanger method. Any differences between UDPS reads and the Sanger sequence were considered UDPS errors. In addition, UDPS has been reported to be more error prone in homopolymeric regions (3 or more identical consecutive nts flanked by non-identical bases) [12]. For this reason, different arrays of mismatch frequencies (μ values) were generated in homopolymeric and non-homopolymeric regions (Table 7).

**Table 7:** Poisson model for quantification of artifactual errors.

Homopolymeric (r = 1)	A	C	T	G
**A**	1	2.34×10^−6^	2.04×10^−6^	3.49×10^−6^
**C**	4.98×10^−6^	1	3.56×10−^5^	1.95×10^−5^
**T**	3.99×10^−6^	2.91×10^−6^	1	2.04×10^−6^
**G**	1.50×10^−4^	3.92×10^−5^	4.40×10^−6^	1

Non-homopolymeric (r = 2)	A	C	T	G
**A**	1	2.93×10^−6^	1.47×10^−6^	7.53×10^−6^
**C**	4.83×10^−6^	1	1.01×10^−5^	3.20×10^−6^
**T**	1.21×10^−6^	7.39×10^−6^	1	1.77×10^−5^
**G**	4.23×10^−5^	1.41×10^−6^	2.91×10^−6^	1

The nucleotide (nt) expected to be found at a given position is expressed as 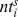, “s” indicates sequence position (s = 1 to 180) and “i” indicates nt of the master sequence found in “s” position (1 = A, 2 = C, 3 = T, 4 = G). An observed variant “s” is represented as 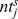, where “j” indicates the nt substitution (1 = A, 2 = C, 3 = T, 4 = G); 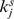 represents the number of occurrences of an “i” to “j” substitution (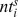 to 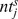) in the N reads covering an “s”. The distribution of mismatch errors was approximated using Poisson distribution, extended to make the parameters depend on the type of mismatch error (12 possible substitutions [A∶C, A∶T, A∶G, C∶A, C∶T, C∶G, T∶A, T∶C, T∶G, G∶A, G∶C, G∶T]) and the region type (homopolymeric/non-homopolymeric). Thus, assuming 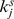 as a Poisson-distributed random variable, the *P*-value associated with this test was calculated as:
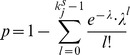
In which λ is the expected number of errors 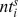 to 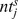 at “s” site: 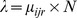 with r referring to the type of region (r = 1 for homopolymeric and r = 2 for non-homopolymeric), with μ_ijr_ values from the array of mismatch frequencies shown in the table. For example, if a T to A change is found (i = 3 and j = 1) at a certain site in a non-homopolymeric region, (r = 2), then 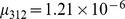 and 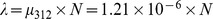. In a type of filter strategy, after applying the Poisson formula, variants with a low probability of being erroneous (*P*<0.05) were included in the analysis.

### Computational analysis

Data accuracy of the UDPS reads was initially validated as recently reported [34]. However, to exclude reads with low-confidence single-base changes, we approximated the distribution of mismatch errors using Poisson distribution [13] instead of an internal control sequence [34] (details in Table 7). Variants with a low probability of being erroneous (*P*<0.05) were included in the analysis.

Longitudinal variability of the HBV quasispecies in the sequentially analyzed MDR patient was assessed by a “blind” algorithm-driven method, which consists of ranking each aa substitution according to the standard deviation (SD) of its frequencies in the validated reads from each of the 5 samples from patient 4, assuming that the substitution with the highest SD value through the 5 consecutive samples would be the most variable.

### Validation of UDPS data

The Poisson-based statistical filter was validated using an independent HBV RT clone, processed as the patient samples. The empirical distribution of mismatch errors in the clone yielded an average of 0.007%, but in 4 positions, errors were higher than 0.02% and lower than 0.03%. Therefore, the sensitivity of UDPS to detect mutations was limited by a mismatch error rate of 0.03%, a value similar to the rate reported using a restriction target sequence as internal control [34]. Nonetheless, to focus the analysis on the most significant changes, the quantifications and biological conclusions reported here are based on mutations comprising ≥0.1% of the viral population.

### Statistical analysis

The frequencies of aa substitutions known to be associated with resistance to NA treatment were compared to the remaining aa changes in the baseline viral populations using the Mann-Whitney *U* test with significance set at a *P*-value of <0.05. The analysis was performed with SPSS, version 15.0 (SPSS Inc., Chicago, USA). Acceptance of a nucleotide substitution was decided by Poisson distribution modeling (details in Table 7).

## Supporting Information

Figure S1 **Three-dimensional representation of the homology model of the HBV polymerase.** The representation of HBV polymerase (yellow) is based on the crystal structure of the catalytic center of the HIV polymerase (black wire) [1], with tenofovir (orange) blocking the active site (YMDD) in blue; in dark grey, the DNA-RNA growing duplex. The most important positions associated with antiviral resistance are identified (rtL180, rtS202, rtM204, and rtV207 in black), as well as the most conserved position detected in our study (rtL155), which is located outside the active site, at the surface of the structure. (Tuske S, Sarafianos SG, Clark AD, Ding J, Naeger LK, et al. (2004) Structures of HIV-1 RT-DNA complexes before and after incorporation of the anti-AIDS drug tenofovir. Nature structural & molecular biology 11: 469–474. Available: http://www.ncbi.nlm.nih.gov/pubmed/15107837. Accessed 2011 Oct 10.)
(PPT)

Table S1 Frequencies of all amino acid substitutions found in the four baseline populations (1 to 4).
(DOC)

Table S2 Comparison of percentage of amino acid changes in the reverse transcriptase and surface coding regions over the four pre-treatment samples.
(DOC)

Dataset S1 Alignment of master nucleotide sequences of the 8 samples analyzed.
(FAS)

Dataset S2 Alignment of master nucleotide sequences of the 8 samples analyzed, translated into amino acids in P open reading frame.
(FAS)

Dataset S3 Alignment of master nucleotide sequences of the 8 samples analyzed, translated into amino acids in S open reading frame. In samples 4C and 4D, the S protein amino acid sequence is truncated at position s172 by the presence of a stop codon, as a result of polymerase substitution rtA181T.
(FAS)
