# Supplementary figures and images for: Ultra-Deep Pyrosequencing Detects Conserved Genomic Sites and Quantifies Linkage of Drug-Resistant Amino Acid Changes in the Hepatitis B Virus Genome
Source: PLoS One. 2012 May 30;7(5):e37874. doi: 10.1371/journal.pone.0037874 (PMC3364280; doi:10.1371/journal.pone.0037874)

## Slide 1
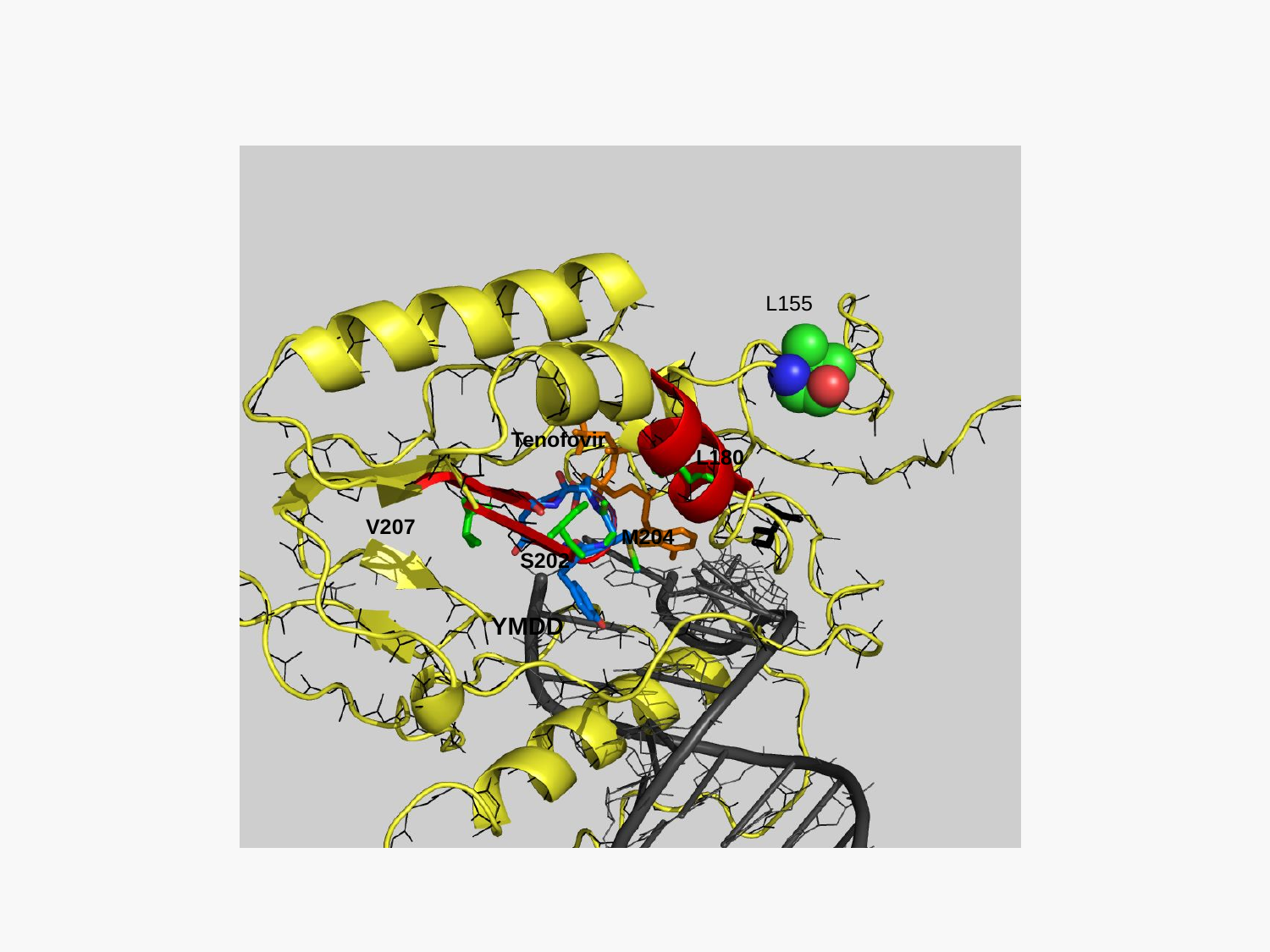

L155
Tenofovir
L180
V207
M204
S202
YMDD

Supplement: Figure S1 — Three-dimensional representation of the homology model of the HBV polymerase. The representation of HBV polymerase (yellow) is based on the crystal structure of the catalytic center of the HIV polymerase (black wire) [1], with tenofovir (orange) blocking the active site (YMDD) in blue; in dark grey, the DNA-RNA growing duplex. The most important positions associated with antiviral resistance are identified (rtL180, rtS202, rtM204, and rtV207 in black), as well as the most conserved position detected in our study (rtL155), which is located outside the active site, at the surface of the structure. (Tuske S, Sarafianos SG, Clark AD, Ding J, Naeger LK, et al. (2004) Structures of HIV-1 RT-DNA complexes before and after incorporation of the anti-AIDS drug tenofovir. Nature structural & molecular biology 11: 469–474. Available: http://www.ncbi.nlm.nih.gov/pubmed/15107837. Accessed 2011 Oct 10.) (PPT) [file pone.0037874.s001.ppt]
