# Supplementary material for: Ultra-Deep Pyrosequencing Detects Conserved Genomic Sites and Quantifies Linkage of Drug-Resistant Amino Acid Changes in the Hepatitis B Virus Genome
Source: PLoS One. 2012 May 30;7(5):e37874. doi: 10.1371/journal.pone.0037874 (PMC3364280; doi:10.1371/journal.pone.0037874)
Supplement: Table S1 — Frequencies of all amino acid substitutions found in the four baseline populations (1 to 4). (DOC) [file pone.0037874.s002.doc]

**Table S1. Frequencies of all amino acid substitutions found in the four baseline populations (1 to 4).**

| **Codon** | **RT**  **aa change** | **Patient (Sample)** | | | | **Mean Frequencies** |
| --- | --- | --- | --- | --- | --- | --- |
|  |  | **1**  **N=32 737** | **2**  **N=13 670** | **3**  **N=62 450** | **4 (4A)**  **N=32 724** |  |
| 148 | Y148C | 0.055 | 0.029 | 0.051 | 0.018 | 0.038 |
| 148 | Y148H | 0.089 | **Master** | 0.086 | 0.037 | 0.071 |
| 148 | Y148Q | 0.000 | 0.015 | 0.000 | 0.000 | 0.004 |
| 148 | Y148R | 0.000 | 0.051 | 0.000 | 0.000 | 0.013 |
| 148 | H148Y | **Master** | **5.048** | **Master** | **Master** | **NC** |
| 148 | Y148* | 0.006 | 0.000 | 0.014 | 0.006 | 0.007 |
| 149 | K149E | 0.046 | 0.044 | 0.046 | 0.015 | 0.038 |
| 149 | K149N | 0.009 | 0.007 | 0.010 | 0.021 | 0.012 |
| 149 | K149Q | 0.021 | 0.022 | 0.021 | 0.006 | 0.018 |
| 149 | K149R | 0.046 | **0.102** | 0.058 | 0.006 | 0.053 |
| 149 | K149T | 0.003 | 0.015 | 0.008 | 0.003 | 0.007 |
| 150 | T150A | **0.113** | **0.161** | 0.090 | 0.037 | **0.100** |
| 150 | T150I | 0.027 | 0.066 | 0.056 | 0.037 | 0.047 |
| 150 | T150P | 0.003 | 0.007 | 0.006 | 0.012 | 0.007 |
| 151 | Y151C | 0.052 | **0.117** | 0.048 | 0.009 | 0.057 |
| 151 | Y151H | 0.046 | 0.037 | 0.030 | 0.009 | 0.030 |
| 151 | Y151* | 0.006 | 0.007 | 0.005 | 0.012 | 0.008 |
| 152 | G152E | 0.040 | 0.029 | 0.067 | 0.043 | 0.045 |
| 152 | G152R | 0.055 | 0.051 | 0.072 | 0.076 | 0.064 |
| 153 | R153C | 0.015 | 0.000 | 0.014 | 0.000 | 0.007 |
| 153 | R153Q | 0.000 | **0.139** | 0.000 | **0.134** | 0.068 |
| 153 | R153W | **Master** | **5.011** | **Master** | 0.055 | **NC** |
| 153 | R153* | **0.128** | 0.015 | **0.101** | 0.000 | 0.061 |
| 154 | K154E | 0.040 | 0.029 | 0.030 | 0.034 | 0.033 |
| 154 | K154N | 0.003 | 0.000 | 0.010 | 0.018 | 0.008 |
| 154 | K154Q | 0.000 | 0.000 | 0.006 | 0.052 | 0.015 |
| 154 | K154R | 0.064 | 0.044 | 0.051 | 0.012 | 0.043 |
| 155 | L155S | 0.021 | 0.007 | 0.014 | 0.000 | 0.011 |
| 156 | H156R | 0.067 | **0.168** | 0.088 | 0.021 | 0.086 |
| 156 | H156Y | 0.049 | 0.080 | 0.053 | 0.018 | 0.050 |
| 157 | L157M | 0.009 | **0.168** | 0.042 | **0.186** | **0.101** |
| 157 | L157P | 0.082 | 0.015 | 0.061 | 0.028 | 0.046 |
| 157 | L157Q | 0.006 | 0.015 | 0.002 | 0.000 | 0.006 |
| 158 | Y158C | 0.018 | 0.037 | 0.016 | 0.000 | 0.018 |
| 158 | Y158H | 0.040 | 0.029 | 0.032 | 0.000 | 0.025 |
| 159 | S159A | 0.003 | 0.044 | 0.002 | 0.009 | 0.014 |
| 159 | S159F | 0.046 | 0.088 | 0.042 | 0.040 | 0.054 |
| 159 | S159P | 0.092 | **0.110** | 0.082 | 0.037 | 0.080 |
| 159 | S159T | 0.012 | 0.073 | 0.003 | 0.006 | 0.024 |
| 160 | H160N | 0.006 | 0.015 | 0.002 | 0.003 | 0.006 |
| 160 | H160R | 0.058 | 0.051 | 0.061 | 0.012 | 0.046 |
| 160 | H160Y | 0.040 | 0.073 | 0.050 | 0.043 | 0.051 |
| 161 | P161L | 0.052 | 0.059 | 0.058 | 0.031 | 0.050 |
| 161 | P161S | 0.043 | 0.029 | 0.064 | 0.037 | 0.043 |
| 161 | P161T | 0.000 | 0.007 | 0.005 | 0.012 | 0.006 |
| 162 | I162L | 0.000 | 0.007 | 0.011 | 0.009 | 0.007 |
| 162 | I162T | 0.092 | **0.110** | 0.086 | 0.079 | 0.092 |
| 162 | I162V | 0.067 | 0.073 | 0.032 | 0.012 | 0.046 |
| 163 | V163A | **0.110** | **0.154** | **0.141** | 0.055 | **0.115** |
| 163 | V163I | 0.082 | **0.154** | 0.056 | 0.098 | 0.097 |
| 164 | L164M | 0.037 | 0.066 | 0.029 | 0.012 | 0.036 |
| 164 | L164P | 0.061 | **0.117** | 0.074 | 0.000 | 0.063 |
| 164 | L164Q | 0.012 | 0.022 | 0.011 | 0.000 | 0.011 |
| 164 | L164R | 0.009 | 0.044 | 0.002 | 0.000 | 0.014 |
| 164 | L164S | 0.000 | 0.000 | 0.000 | 0.012 | 0.003 |
| 164 | L164W | 0.000 | 0.000 | 0.000 | 0.012 | 0.003 |
| 165 | G165D | 0.064 | 0.059 | 0.045 | 0.028 | 0.049 |
| 165 | G165S | 0.089 | 0.051 | 0.056 | 0.070 | 0.067 |
| 166 | F166L | **0.125** | **0.198** | **0.122** | 0.046 | **0.123** |
| 166 | F166S | 0.043 | 0.022 | 0.038 | 0.006 | 0.027 |
| 167 | R167C | 0.067 | 0.044 | 0.066 | 0.055 | 0.058 |
| 167 | R167H | 0.086 | **0.117** | 0.070 | 0.064 | 0.084 |
| 167 | R167P | 0.000 | 0.015 | 0.003 | 0.003 | 0.005 |
| 168 | K168E | 0.027 | 0.022 | 0.026 | 0.003 | 0.020 |
| 168 | K168N | 0.015 | 0.007 | 0.002 | 0.003 | 0.007 |
| 168 | K168R | 0.064 | 0.051 | 0.038 | 0.003 | 0.039 |
| 168 | K168T | 0.000 | 0.015 | 0.002 | 0.000 | 0.004 |
| 169 | I169L | 0.012 | 0.000 | 0.002 | 0.031 | 0.011 |
| 169 | I169M | 0.040 | 0.037 | 0.034 | 0.021 | 0.033 |
| 169 | I169T | 0.015 | 0.000 | 0.010 | 0.000 | 0.006 |
| 169 | I169V | 0.061 | 0.051 | 0.059 | 0.031 | 0.051 |
| 170 | P170L | 0.031 | 0.022 | 0.043 | 0.043 | 0.035 |
| 170 | P170S | 0.040 | 0.051 | 0.040 | 0.021 | 0.038 |
| 171 | M171I | 0.073 | 0.095 | 0.086 | 0.098 | 0.088 |
| 171 | M171K | 0.000 | 0.015 | 0.000 | 0.000 | 0.004 |
| 171 | M171T | 0.040 | 0.088 | 0.042 | 0.015 | 0.046 |
| 171 | M171V | 0.043 | 0.029 | 0.026 | 0.012 | 0.027 |
| 172 | G172E | 0.031 | 0.095 | 0.056 | 0.067 | 0.062 |
| 172 | G172R | 0.061 | 0.059 | 0.053 | 0.070 | 0.061 |
| 172 | G172V | 0.000 | 0.015 | 0.002 | 0.000 | 0.004 |
| 173 | V173A | 0.092 | **0.102** | 0.072 | 0.012 | 0.070 |
| 173 | V173L | 0.012 | 0.007 | 0.006 | 0.049 | 0.019 |
| 173 | V173M | 0.086 | 0.095 | 0.099 | 0.073 | 0.088 |
| 174 | G174C | 0.009 | 0.015 | 0.002 | 0.000 | 0.006 |
| 174 | G174D | 0.064 | 0.037 | 0.067 | 0.067 | 0.059 |
| 174 | G174S | 0.061 | 0.051 | 0.067 | 0.028 | 0.052 |
| 175 | L175F | 0.052 | 0.066 | 0.053 | 0.024 | 0.049 |
| 175 | L175I | 0.009 | 0.000 | 0.014 | 0.012 | 0.009 |
| 175 | L175P | 0.079 | **0.110** | 0.069 | 0.040 | 0.074 |
| 175 | L175R | 0.012 | 0.015 | 0.006 | 0.000 | 0.008 |
| 176 | S176G | 0.031 | 0.066 | 0.030 | 0.006 | 0.033 |
| 176 | S176N | 0.043 | 0.051 | 0.048 | 0.049 | 0.048 |
| 177 | P177L | 0.049 | 0.080 | 0.048 | 0.043 | 0.055 |
| 177 | P177S | 0.027 | 0.066 | 0.037 | 0.031 | 0.040 |
| 178 | F178L | **0.116** | **0.161** | **0.118** | 0.070 | **0.116** |
| 178 | F178S | 0.055 | 0.073 | 0.054 | 0.028 | 0.053 |
| 178 | F178V | 0.003 | 0.000 | 0.011 | 0.000 | 0.004 |
| 179 | L179F | 0.049 | 0.037 | 0.045 | 0.040 | 0.043 |
| 179 | L179P | **0.116** | **0.132** | 0.067 | 0.049 | 0.091 |
| 180 | L180S | 0.052 | 0.095 | 0.059 | 0.009 | 0.054 |
| 181 | A181S | 0.003 | 0.000 | 0.018 | 0.018 | 0.010 |
| 181 | A181T | 0.073 | **0.176** | 0.070 | 0.089 | **0.102** |
| 181 | A181V | 0.027 | 0.029 | 0.030 | 0.049 | 0.034 |
| 182 | Q182R | 0.024 | 0.022 | 0.018 | 0.006 | 0.018 |
| 182 | Q182* | 0.058 | 0.051 | 0.030 | 0.018 | 0.040 |
| 183 | F183C | 0.003 | 0.015 | 0.003 | 0.000 | 0.005 |
| 183 | F183L | **0.107** | **0.110** | 0.074 | 0.043 | 0.083 |
| 183 | F183S | 0.055 | 0.080 | 0.024 | 0.003 | 0.041 |
| 184 | T184A | 0.067 | 0.073 | 0.058 | 0.006 | 0.051 |
| 184 | T184I | 0.043 | 0.066 | 0.051 | 0.031 | 0.048 |
| 184 | T184P | 0.009 | 0.022 | 0.000 | 0.000 | 0.008 |
| 185 | S185G | 0.092 | 0.095 | 0.056 | 0.024 | 0.067 |
| 185 | S185N | 0.043 | 0.088 | 0.058 | 0.049 | 0.059 |
| 186 | A186G | 0.000 | 0.015 | 0.003 | 0.003 | 0.005 |
| 186 | A186T | 0.086 | 0.088 | 0.051 | 0.061 | 0.071 |
| 186 | A186V | 0.040 | **0.102** | 0.056 | 0.046 | 0.061 |
| 187 | I187L | 0.046 | **5.026** | 0.006 | 0.028 | **1.276** |
| 187 | I187M | 0.000 | 0.022 | 0.000 | 0.000 | 0.005 |
| 187 | I187T | 0.055 | 0.029 | 0.054 | 0.018 | 0.039 |
| 187 | I187V | 0.082 | 0.073 | 0.018 | 0.009 | 0.046 |
| 188 | C188R | 0.034 | 0.037 | 0.019 | 0.003 | 0.023 |
| 188 | C188Y | 0.046 | 0.080 | 0.040 | 0.028 | 0.048 |
| 189 | S189L | 0.015 | 0.059 | 0.038 | 0.034 | 0.036 |
| 189 | S189P | 0.073 | **0.117** | 0.061 | 0.028 | 0.070 |
| 190 | V190A | 0.067 | 0.080 | 0.069 | 0.012 | 0.057 |
| 190 | V190M | 0.055 | 0.080 | 0.061 | 0.083 | 0.070 |
| 191 | V191A | 0.064 | 0.080 | 0.086 | 0.040 | 0.068 |
| 191 | V191I | **0.107** | 0.080 | **0.131** | **0.590** | **0.227** |
| 191 | V191L | 0.003 | 0.007 | 0.000 | 0.018 | 0.007 |
| 192 | R192C | 0.055 | 0.080 | 0.042 | 0.043 | 0.055 |
| 192 | R192H | 0.037 | **0.124** | 0.059 | 0.061 | 0.070 |
| 193 | R193G | 0.076 | 0.095 | 0.034 | 0.006 | 0.053 |
| 193 | R193K | 0.082 | 0.088 | 0.074 | 0.070 | 0.079 |
| 193 | R193M | 0.006 | 0.015 | 0.000 | 0.000 | 0.005 |
| 194 | A194D | 0.012 | 0.000 | 0.003 | 0.000 | 0.004 |
| 194 | A194T | 0.067 | **0.241** | 0.074 | 0.067 | **0.112** |
| 194 | A194V | 0.058 | 0.080 | 0.038 | 0.049 | 0.056 |
| 195 | F195L | 0.040 | 0.029 | 0.026 | 0.006 | 0.025 |
| 195 | F195S | 0.046 | 0.088 | 0.058 | 0.018 | 0.052 |
| 196 | P196H | 0.003 | 0.015 | 0.002 | 0.003 | 0.006 |
| 196 | P196L | 0.055 | 0.029 | 0.050 | 0.024 | 0.040 |
| 196 | P196S | 0.043 | 0.073 | 0.066 | 0.024 | 0.052 |
| 197 | H197N | 0.003 | 0.000 | 0.013 | 0.000 | 0.004 |
| 197 | H197R | **0.110** | 0.095 | 0.064 | 0.021 | 0.073 |
| 197 | H197Y | 0.064 | 0.066 | 0.082 | 0.046 | 0.064 |
| 198 | C198R | 0.049 | **0.110** | 0.042 | 0.012 | 0.053 |
| 198 | C198Y | 0.052 | 0.051 | 0.042 | 0.061 | 0.051 |
| 199 | L199F | 0.015 | 0.000 | 0.000 | 0.000 | 0.004 |
| 199 | L199S | 0.082 | 0.080 | 0.062 | 0.037 | 0.066 |
| 199 | L199V | 0.021 | **3.980** | 0.003 | 0.009 | **1.003** |
| 199 | L199W | 0.000 | 0.037 | 0.002 | 0.000 | 0.010 |
| 200 | A200T | 0.037 | **0.161** | 0.072 | 0.046 | 0.079 |
| 200 | A200V | 0.034 | 0.066 | 0.043 | 0.024 | 0.042 |
| 201 | F201L | 0.043 | 0.088 | 0.040 | 0.009 | 0.045 |
| 201 | F201S | 0.058 | 0.044 | 0.053 | 0.003 | 0.039 |
| 202 | S202G | 0.058 | 0.015 | 0.072 | 0.003 | 0.037 |
| 202 | S202N | 0.046 | 0.088 | 0.051 | 0.043 | 0.057 |
| 203 | Y203C | 0.027 | 0.022 | 0.043 | 0.003 | 0.024 |
| 203 | Y203H | 0.058 | 0.015 | 0.038 | 0.006 | 0.029 |
| 204 | M204I | 0.067 | **0.351** | 0.086 | 0.079 | **0.146** |
| 204 | M204R | 0.015 | 0.000 | 0.011 | 0.006 | 0.008 |
| 204 | M204T | 0.055 | 0.059 | 0.038 | 0.006 | 0.040 |
| 204 | M204V | 0.058 | 0.088 | 0.045 | 0.021 | 0.053 |
| 205 | D205G | 0.031 | 0.037 | 0.038 | 0.006 | 0.028 |
| 205 | D205N | 0.082 | **0.256** | 0.054 | 0.046 | **0.110** |
| 206 | D206E | 0.012 | 0.000 | 0.003 | 0.003 | 0.005 |
| 206 | D206G | **0.101** | 0.080 | 0.058 | 0.015 | 0.064 |
| 206 | D206N | 0.052 | **0.132** | 0.058 | 0.024 | 0.066 |
| 207 | V207A | **0.104** | **0.102** | 0.085 | 0.040 | 0.083 |
| 207 | V207I | 0.009 | 0.015 | **0.219** | 0.009 | 0.063 |
| 207 | V207L | 0.021 | 0.029 | 0.011 | 0.018 | 0.020 |
| 207 | V207M | **0.116** | **0.124** | **0.107** | **0.128** | **0.119** |
| 208 | V208A | 0.049 | 0.051 | 0.043 | 0.012 | 0.039 |
| 208 | V208I | 0.049 | **0.205** | 0.058 | 0.055 | 0.092 |

aa, amino acid; NC, not-calculated; RT, reverse transcriptase

Master = changes that appear in the master sequence in any of the baseline populations.

Frequencies of the most prevalent amino acid substitutions (≥0.1%) are shown in bold and those below mismatch error rate (0.03%) are shown in grey. The mean frequency of each amino acid substitution in the four baseline populations was calculated, the mean frequencies ≥0.1% (indicating the most significant substitutions) are shown in bold and shaded.

On linkage analysis, no combinations of polymerase variants were detected in any of the baseline populations.
