# Supplementary material for: Ultra-Deep Pyrosequencing Detects Conserved Genomic Sites and Quantifies Linkage of Drug-Resistant Amino Acid Changes in the Hepatitis B Virus Genome
Source: PLoS One. 2012 May 30;7(5):e37874. doi: 10.1371/journal.pone.0037874 (PMC3364280; doi:10.1371/journal.pone.0037874)
Supplement: Table S2 — Comparison of percentage of amino acid changes in the reverse transcriptase and surface coding regions over the four pre-treatment samples. (DOC) [file pone.0037874.s003.doc]

**Table S2. Comparison of percentage of amino acid changes in the reverse transcriptase and surface coding regions over the four pre-treatment samples.**

| **Polymerase**  **residue** | **Surface**  **residue** | **Polymerase** | | | **Surface** | | |
| --- | --- | --- | --- | --- | --- | --- | --- |
|  |  | **(%) Total changes** | **(%) dn** | ***dn/ds*** | **(%) Total changes** | **(%) dn** | ***dn/ds*** |
| **Y148** | **T140** | 1.46 | 1.39 | *19.39* | 0.19 | 0.14 | *2.77* |
| **K149** | **K141** | 0.16 | 0.13 | *4.67* | 0.21 | 0.11 | *1.12* |
| **T150** | **P142** | 0.21 | 0.16 | *3.16* | 0.13 | 0.10 | *3.14* |
| **Y151** | **T143** | 0.18 | 0.10 | *1.22* | 0.22 | 0.15 | *2.22* |
| **G152** | **D144** | 0.19 | 0.11 | *1.52* | 1.43 | 0.14 | *0.10* |
| **W153** | **G145** | 1.53 | 1.44 | *16.98* | 0.27 | 0.22 | *4.47* |
| **K154** | **N146** | 0.14 | 0.11 | *2.66* | 0.14 | 0.10 | *2.35* |
| **L155** | **C147** | 0.14 | 0.02 | *0.16* | 0.14 | 0.09 | *1.89* |
| **H156** | **T148** | 0.19 | 0.14 | *3.17* | 0.35 | 0.14 | *0.65* |
| **L157** | **C149** | 0.33 | 0.16 | *0.94* | 0.15 | 0.12 | *4.84* |
| **Y158** | **I150** | 0.12 | 0.05 | *0.67* | 0.21 | 0.11 | *1.03* |
| **S159** | **P151** | 0.21 | 0.17 | *4.56* | 0.15 | 0.09 | *1.60* |
| **H160** | **I152** | 0.17 | 0.11 | *1.78* | 0.16 | 0.11 | *2.23* |
| **P161** | **P153** | 0.17 | 0.11 | *1.71* | 0.17 | 0.12 | *2.15* |
| **I162** | **S154** | 0.27 | 0.15 | *1.29* | 0.32 | 0.22 | *2.19* |
| **V163** | **S155** | 0.30 | 0.22 | *2.54* | 0.36 | 0.20 | *1.34* |
| **L164** | **W156** | 0.35 | 0.14 | *0.63* | 0.29 | 0.27 | *11.00* |
| **G165** | **A157** | 0.18 | 0.12 | *2.29* | 7.00 | 0.11 | *0.02* |
| **F166** | **F158** | 0.21 | 0.16 | *2.97* | 0.15 | 0.09 | *1.62* |
| **R167** | **A159** | 0.25 | 0.15 | *1.59* | 0.21 | 0.19 | *8.63* |
| **K168** | **K160** | 0.10 | 0.07 | *2.72* | 0.14 | 0.09 | *1.81* |
| **I169** | **Y161** | 0.48 | 0.10 | *0.27* | 0.46 | 0.42 | *11.05* |
| **P170** | **L162** | 0.14 | 0.08 | *1.50* | 0.12 | 0.06 | *1.00* |
| **M171** | **W163** | 0.18 | 0.17 | *16.67* | 0.21 | 0.20 | *22.98* |
| **G172** | **E164** | 0.27 | 0.13 | *0.93* | 0.31 | 0.22 | *2.55* |
| **V173** | **W165** | 0.27 | 0.18 | *2.14* | 0.23 | 0.22 | *15.91* |
| **G174** | **A166** | 0.20 | 0.12 | *1.45* | 0.20 | 0.14 | *2.47* |
| **L175** | **S167** | 0.23 | 0.15 | *1.82* | 0.20 | 0.17 | *5.11* |
| **S176** | **V168** | 0.22 | 0.09 | *0.67* | 0.23 | 0.19 | *4.17* |
| **P177** | **R169** | 0.19 | 0.10 | *1.21* | 0.26 | 0.14 | *1.20* |
| **F178** | **F170** | 0.27 | 0.18 | *2.02* | 0.20 | 0.16 | *3.67* |
| **L179** | **S171** | 0.20 | 0.14 | *2.36* | 0.24 | 0.16 | *1.94* |
| **L180** | **W172** | 0.24 | 0.07 | *0.41* | 0.28 | 0.26 | *13.00* |
| **A181** | **L173** | 0.27 | 0.15 | *1.26* | 0.20 | 0.16 | *4.00* |
| **Q182** | **S174** | 0.17 | 0.06 | *0.61* | 0.21 | 0.13 | *1.58* |
| **F183** | **L175** | 0.19 | 0.13 | *2.62* | 0.16 | 0.07 | *0.75* |
| **T184** | **L176** | 0.16 | 0.11 | *2.14* | 0.17 | 0.06 | *0.48* |
| **S185** | **V177** | 0.26 | 0.14 | *1.16* | 0.26 | 0.19 | *2.59* |
| **A186** | **P178** | 0.20 | 0.14 | *2.63* | 1.45 | 0.12 | *0.09* |
| **I187** | **F179** | 1.43 | 1.36 | *19.30* | 0.13 | 0.11 | *4.48* |
| **C188** | **V180** | 0.18 | 0.07 | *0.69* | 0.23 | 0.16 | *2.19* |
| **S189** | **Q181** | 0.19 | 0.11 | *1.49* | 0.19 | 0.12 | *1.70* |
| **V190** | **W182** | 0.20 | 0.13 | *1.89* | 0.37 | 0.34 | *11.33* |
| **V191** | **F183** | 0.38 | 0.30 | *3.99* | 0.20 | 0.15 | *2.69* |
| **R192** | **V184** | 0.20 | 0.13 | *1.73* | 0.21 | 0.15 | *2.37* |
| **R193** | **G185** | 0.22 | 0.14 | *1.73* | 0.28 | 0.16 | *1.38* |
| **A194** | **L186** | 0.32 | 0.18 | *1.22* | 0.23 | 0.21 | *10.29* |
| **F195** | **S187** | 0.13 | 0.08 | *1.62* | 0.17 | 0.11 | *1.90* |
| **P196** | **P188** | 0.16 | 0.10 | *1.86* | 0.17 | 0.10 | *1.43* |
| **H197** | **T189** | 0.23 | 0.15 | *1.81* | 0.22 | 0.16 | *2.64* |
| **C198** | **V190** | 0.21 | 0.12 | *1.31* | 1.21 | 0.15 | *0.14* |
| **L199** | **W191** | 1.23 | 1.09 | *7.44* | 0.25 | 0.23 | *10.21* |
| **A200** | **L192** | 0.26 | 0.13 | *1.00* | 0.22 | 0.17 | *3.71* |
| **F201** | **S193** | 0.18 | 0.09 | *0.94* | 0.17 | 0.14 | *3.55* |
| **S202** | **A194** | 0.19 | 0.10 | *1.14* | 0.18 | 0.15 | *4.94* |
| **Y203** | **I195** | 0.11 | 0.06 | *1.10* | 0.15 | 0.13 | *8.35* |
| **M204** | **W196** | 0.25 | 0.24 | *26.37* | 0.31 | 0.30 | *21.37* |
| **D205** | **M197** | 0.18 | 0.14 | *3.85* | 0.14 | 0.13 | *16.31* |
| **D206** | **M198** | 0.21 | 0.14 | *1.88* | 0.35 | 0.33 | *17.52* |
| **V207** | **W199** | 0.41 | 0.29 | *2.35* | 0.36 | 0.34 | *20.50* |
| **V208** | **Y200** | 0.22 | 0.14 | *1.68* | 0.21 | 0.16 | *3.68* |

(%) dn = Percentage of the total nucleotide changes that are non-synonymous (implies an amino acid change)

dn/ds = Ratio of non-synonymous (dn) versus synonymous (ds) nucleotide changes

Each position of one coding region overlaps with two positions of the other. To simplify depiction of the influence of one open reading frame over the other, this table shows only one of the overlapping patterns: the first nucleotide of a polymerase (P) codon affects the third nucleotide of the overlapping surface (S) codon (P_1_/S_3_). In this pattern, each overlapping residue of the P and S open reading frames is presented in order of codon position to enable comparison of the ratio of non-synonymous to synonymous nucleotide changes. Results from the other pattern (P_2-3_/S_1-2_) were comparable.
